# Cryogels: recent applications in 3D-bioprinting, injectable cryogels, drug delivery, and wound healing

**DOI:** 10.3762/bjoc.17.171

**Published:** 2021-10-14

**Authors:** Luke O Jones, Leah Williams, Tasmin Boam, Martin Kalmet, Chidubem Oguike, Fiona L Hatton

**Affiliations:** 1 Department of Materials, Loughborough University, Loughborough, LE11 3TU, UK

**Keywords:** 3D-bioprinting, cryogels, drug delivery, injectable cryogel, macroporous hydrogel, wound healing

## Abstract

Cryogels are macroporous polymeric structures formed from the cryogelation of monomers/polymers in a solvent below freezing temperature. Due to their inherent interconnected macroporosity, ease of preparation, and biocompatibility, they are increasingly being investigated for use in biomedical applications such as 3D-bioprinting, drug delivery, wound healing, and as injectable therapeutics. This review highlights the fundamentals of macroporous cryogel preparation, cryogel properties that can be useful in the highlighted biomedical applications, followed by a comprehensive review of recent studies in these areas. Research evaluated includes the use of cryogels to combat various types of cancer, for implantation without surgical incision, and use as highly effective wound dressings. Furthermore, conclusions and outlooks are discussed for the use of these promising and durable macroporous cryogels.

## Introduction

Gels can be defined as polymer networks that are expanded throughout their whole volume by a fluid. In the case of hydrogels, the network component is a hydrophilic polymer, and the swelling agent is water [1]. However, their lack of interconnected macropores and elasticity, required properties for a variety of biomedical applications, has demanded the development of cryogels. Cryogels are a class of hydrogels formed below the freezing point of the solvent. Following a cycle of freeze–thawing, a supermacroporous interconnected structure is formed [2]. The uniform porous structure facilitates cell proliferation and waste exchange, e.g., in a scaffold for tissue engineering applications [3]. Novel biomedical and biotechnical applications of cryogels can also be found in controlled drug delivery, carriers for cell immobilization, sensors, bioseparation, purification, and wound dressing [4–6]. Here we discuss the preparation of cryogels, their properties and applications, focussing on recent reports of cryogels in emerging applications, including bioprinting, injectable cryogels, drug delivery, and wound healing, as investigations in these key areas have intensified in recent years. Previous reviews discuss the biomedical applications of cryogels, specifically composite cryogels [7] and biodegradable cryogels [8], while injectable cryogels have also been reviewed by Eggermont et al. [9] and Çimen et al. [10]. Here, we provide an update on the most recent literature relating to drug delivery and injectable cryogels, and we discuss 3D printing and wound healing in detail.

## Review

### 1. Cryogel synthesis

**Figure 1:**
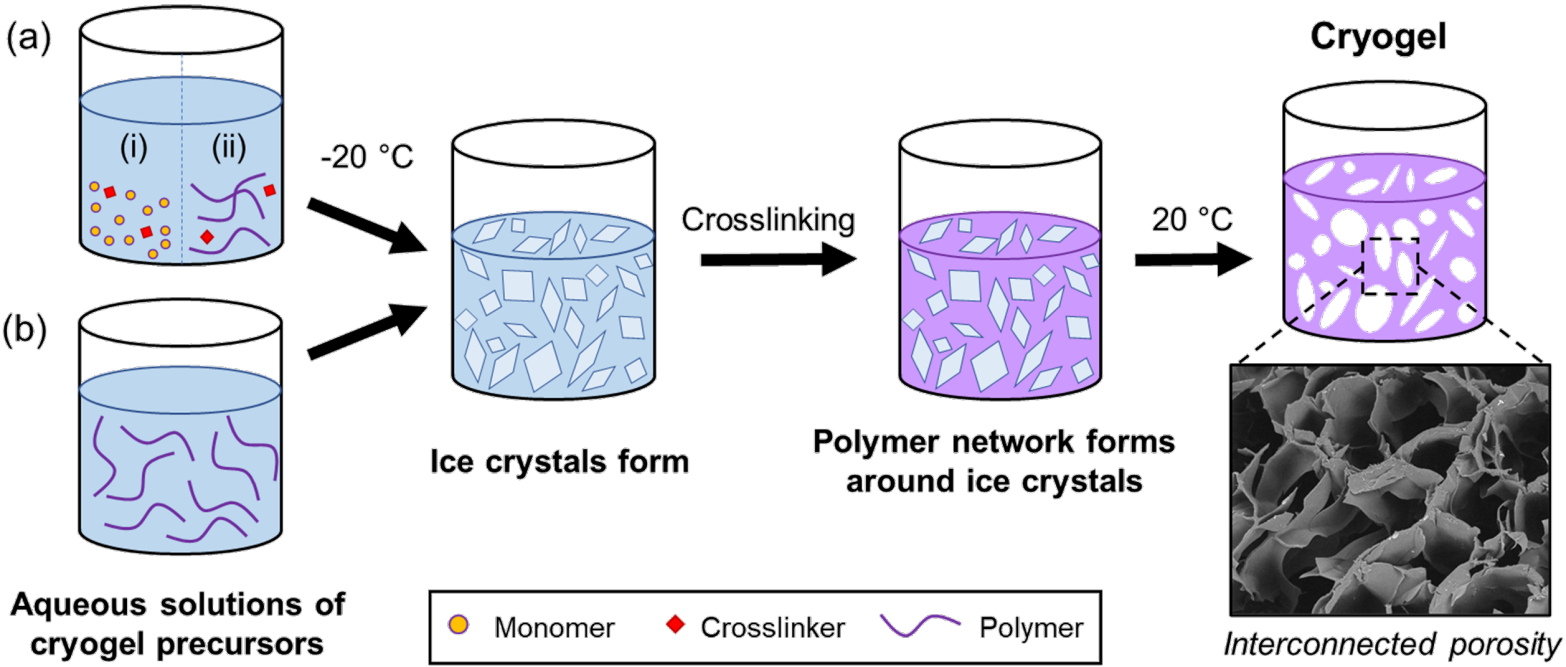
Schematic representation of the process of aqueous cryogel formation, using (a) monomers/small molecule (i) or polymeric precursors (ii) to form chemically crosslinked cryogels, or (b) polymeric precursors to form physically crosslinked cryogels. Temperatures are indicative of those typically used for aqueous cryogel formation.

Cryogels are produced by a cryogelation process, see Figure 1. While this process is relatively universal, differences exist in the initial materials used, hence resulting in either chemically or physically crosslinked cryogels [11–13]. Firstly, a reaction mixture is prepared consisting of monomers/small molecule precursors, polymeric precursors, or a combination of the two (Figure 1) and solvent (e.g., water). ). It is worth noting that while we refer to water as the solvent here and in Figure 1, other solvents can be used, providing they have an appropriate melting/freezing temperature. Typically, the reagents will comprise only 5–20% of the reaction mixture. Often an initiator may also be required to initiate the polymerisation of the monomers/small molecule precursors. The solution is then cooled to below the freezing point of water, whereby ice crystals form. Importantly, here the solvent (water) solidifies (freezes) and forms solid solvent porogens within the structure allowing the polymer network to form around these crystals, templating the porous structure of the final cryogel. In chemically crosslinked cryogels (Figure 1a), the monomers polymerise and crosslink (in the presence of a crosslinker) around the ice crystals to form a network. In physically crosslinked cryogels (Figure 1b), once the solution is cooled below the freezing temperature the polymeric precursors can form chain entanglements and/or crystalline regions to form physical crosslinks between the chains. After thawing, the cryogel is formed and porous structure is retained. Often in physically crosslinked systems more than one freeze–thaw cycle is required to result in a free-standing cryogel. Common cryogel compositions include natural polymers such as gelatin and chitosan, and synthetic acrylamide-based polymers and poly(vinyl alcohol) (PVA) [12–14]. The reader is directed to a recent review by Thakor and co-workers which discusses cryogel synthesis in greater depth [15].

### 2. Cryogel properties

Cryogels are macroporous hydrogels with interconnected porosity, with high swelling capacities and large surface areas. Ultimately, many of the final cryogel properties are dependent on the choice of polymer/monomer composition used. However, other factors such as crosslinking, pore size, wall thickness, and incorporation of fillers or additives also affect the cryogel properties. Cryogel wall thickness and density, pore size and pore size distribution can be influenced by the method used for preparation, for example, by increasing the freezing rate smaller pores can be observed [13]. Final cryogel properties including biocompatibility, mechanical and thermal properties, and degradability are influenced by a variety of factors. Perhaps the most important factor is the chemical composition as this determines whether the cryogel is biocompatible or degradable and to some extent influences the mechanical and thermal properties of the cryogel. Mechanical properties are mainly influenced by porosity and degree of crosslinking, while crosslinking also influences biocompatibility and degradability. In chemically crosslinked cryogels the mechanical properties can be influenced by the degree of crosslinking (ratio of monomer to crosslinking agent), while the degree of crosslinking is tuned in physically crosslinked cryogels by varying the number of freeze–thaw cycles.

Pore size, wall thickness, and wall density are of significant importance for cryogels properties [16]. Thicker walls and higher wall densities typically result in improved mechanical properties and are influenced by the concentration of monomer or precursors used as well as the type of crosslinking in the cryogel. Moreover, the processing conditions used when synthesising cryogels have a vast effect on the internal structure. An accelerated freezing rate or reduction in cryogelation temperature will lead to smaller pore sizes throughout the cryogel. This is because the solvent freezes at a faster rate, allowing only a small amount of crystal growth [13–14]. Ivanov et al. researched the production of poly(acrylamide) (PAAm) cryogels and found that reducing the freezing temperature by 15 °C caused an average 30 μm decrease in pore size diameter [14,17]. In contrast, it has been reported that with cryogels crosslinked with glutaraldehyde, the freezing temperature does not affect the pore size [13].

**Figure 2:**
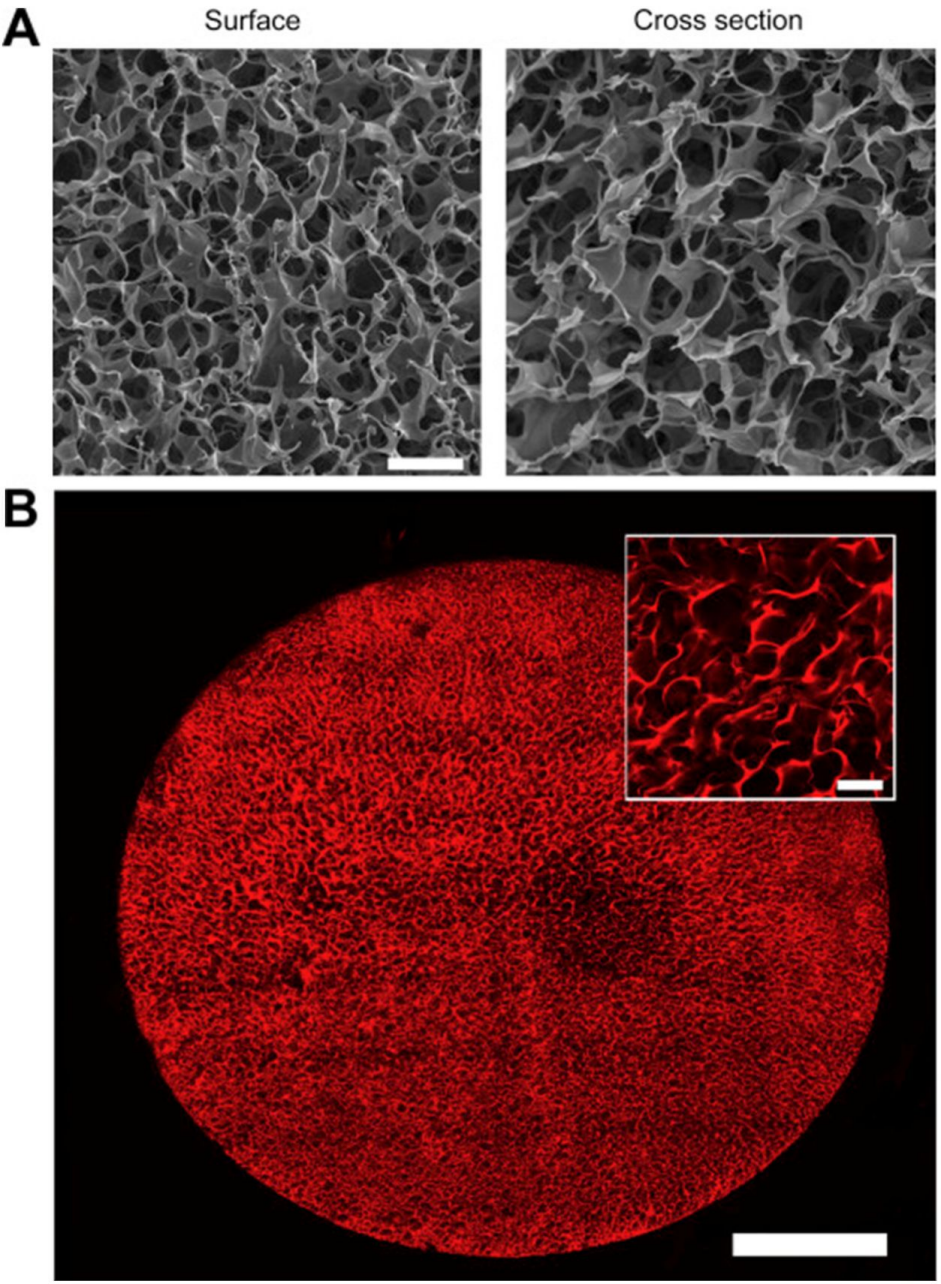
Microarchitecture of gelatin cryogels. (A) Surface and cross-sectional SEM micrographs of highly porous 1.0% (w/v) gelatin cryogels (scale bar = 50 μm). (B) 2-photon imaging at a depth of 150 μm below the surface of a rhodamine-gelatin cryogel (scale bar = 1 mm). The inset shows a magnified view at the centre of the scaffold diameter (scale bar = 100 μm). Images are representative of at least 5 gels imaged using each modality. Reprinted from [18], Biomaterials, vol. 35, issue 8, by S. T. Koshy; T. C. Ferrante; S. A. Lewin; D. J. Mooney, “Injectable, porous, and cell-responsive gelatin cryogels”, 2477–2487, Copyright (2014), with permission from Elsevier. This content is not subject to CC BY 4.0.

An additional consideration is that during cryogelation a temperature gradient will be present. The exterior of the sample will be first exposed to the cold temperature, leading to enhanced freezing rates and smaller pore sizes compared to the inner cryogel material, resulting in a heterogeneous pore size distribution, see Figure 2 [18]. This has led to concerns over producing cryogels with dimensions greater than 25 mm [19]. Although, a report by Macková et al. outlines the benefits of graduated pore size distribution in hydrogels used in tissue engineering, since many human body tissues also exhibit a heterogeneous morphology [20]. This finding was further reiterated by Sen et al., who also explored heterogeneous pore size distribution for tissue engineering applications [21]. Cryostructuring, including directional freezing of cryogels, has been used to achieve varying degrees of porosity and aligned porosity or anisotropy within cryogels. This approach to cryogel preparation has recently been discussed in a review by Shiekh et al. [22] where different cryogel formats were also considered.

Chemical crosslinking can provide satisfying and tailored mechanical properties, however toxic compounds are used as crosslinking agents, which can be difficult to extract and can impair the biocompatibility [4]. Instead, with physical crosslinking, no organic solvents or toxic crosslinking agents are used, therefore no danger of residue left in the final material, which makes this method highly important for biomedical applications [4–5]. As the production is easier, it also results in lower cost. According to Zhang et al., the challenge has been to obtain satisfactory properties without any chemical modification, while retaining the biocompatibility, biodegradability, and bioactivity [4]. However, Bagri et al. confirm that physically crosslinked PVA cryogels show even greater mechanical strength than their chemically crosslinked counterparts [23]. Therefore, it is achievable to obtain similar properties with physical crosslinking, yet the matter of reproducibility and scalability remains an issue.

To assess the mechanical performance of cryogels, compression testing is frequently carried out [12–13]. It has been reported that 99.8% compression can be achieved on continual occasions without any adverse effects, though this was for cryogels based on silk fibroin which is known for its elastic nature [14,24]. In contrast, other reports have cited lower compressibility values in the region of 50–75% [12,25]. It has been found that decreasing the pore size will increase the compressive strength [26], though in contrast to this Dispinar et al. found that increased porosity resulted in higher compressive stresses being achieved. Further to this, compressive strain was found to increase with porosity [25], suggesting that the brittle nature observed for some hydrogels may be due to their lack of macropores [27–28]. One such application where a low compressive strain would not be desirable is if the cryogel was to be injectable (see section 5.2). It has therefore been suggested that the ideal porosity for injectable cryogels is 91%. This figure was the suggested value for cryogels composed of methacrylated gelatin, therefore the value will change slightly with different chemical constituents [18].

For various applications of cryogels, degradation of the material is required, yet in many cases the cryogel is still required to perform some functionality during degradation. Therefore, it would be beneficial if the mechanical properties of the cryogel were measured throughout its degradation [25,29]. It has been suggested that during degradation of cryogels, the walls of the cryogel decrease in thickness and are in some cases broken. This analysis was made for enzyme-degraded cryogels, so it is unclear whether the process is likely to occur for cryogels degraded by other mechanisms such as disulphide cleavage [30–31] and hydrolysis [8]. It has also been found that degradation of chitosan/dextran cryogels resulted in an average increase in pore size, possibly due to thinning of the pore walls and reduction in crosslinks [32]. In general, mechanical property analysis of degraded cryogels is a topic largely overlooked by current literature, despite the importance of it for applications such as scaffold materials [29]. A recent review article by Savina et al. highlights biodegradable cryogels and their applications, including a variety of biocompatible polysaccharide-based cryogels [8].

### 3. Stimuli-responsive cryogels

Stimuli-responsive properties are often desirable for biomaterials used in drug-delivery applications. Here, we focus on temperature and solution pH response of cryogels as detailed below.

#### 3.1. Temperature-responsive cryogels

This is a property which can easily be applied and manipulated through careful polymer selection. For example, altering the ratio of hydrophobic and hydrophilic polymers in the final cryogel structure can allow for fine tuning of the responsive behaviour [33–34].

At specific and unique temperatures, phase changes occur to a temperature-responsive polymer, physically changing the properties and/or morphology. Often it can be characterised in terms of swelling, as the solubility of a polymer within a solvent, or solvation state, changes with temperature in thermally responsive cryogels. This leads to variation in cryogel volume, as at differing temperatures the nature of intra- and intermolecular hydrogen bonding changes, leading to variations on how hydrated the cryogel is, triggering a volume phase transition [34–35]. Changes in solubility can be described by the upper critical solution temperature (UCST) and lower critical solution temperatures (LCST). The UCST is the temperature at which a polymer becomes soluble upon heating, and the LCST is the temperature at which polymers become insoluble upon heating. Any LCST or UCST behaviour can be identified from a polymer/solvent phase diagram, if it has both one-phase and two-phase regions [34,36].

Most commonly, the physical change in properties induced is used when transferring from room temperature to another environment (i.e., body temperature). This leads to potential applications such as injectable biodegradable scaffolds in tissue engineering, or utilising the changing surface properties for in vitro cell culture applications [36–38]. Furthermore, a polymer in cryogel form which exhibits LCST behaviour at below the body temperature of ≈37 °C would be suitable to use for medicinal applications in humans, as it would be insoluble at above these temperatures (i.e., normal body environment) and so would retain its structure when introduced to the human body, and not degrade or dissolve straight away. Poly(*N*-isopropylacrylamide) (PNIPAM) is a well-known example of a thermo-responsive polymer, which exhibits a phase transition close to body temperature and has been used in cryogels to infer temperature responsive behaviour [11,33,39]. Thermoresponsive cryogels comprising oligoethylene glycol have also been reported with dual shape memory behaviour [40]. Natural polymers such as cellulose derivatives, chitosan, gelatin, and dextran exhibit temperature-responsive properties and have been used in cryogels.

#### 3.2. pH-Responsive cryogels

The degree of swelling is affected by the chemical composition of the cryogel, and the nature of the medium, such as pH, ionic strength, and swelling medium composition [34,41]. This affects the interactions of pH-sensitive polymers (both polymer–solvent and polymer–polymer interactions), as functional groups on the polymer chains can have weak acidic characteristics if they release protons, or weak basic characteristics if they accept protons, in response to changes in pH. This can come about from even small variations in solution pH, depending on the degree of ionisation and p*K*_a_ value (index to express acidity of weak acids) of the polymer [35]. Polymers responsive to pH can be classified by the functional groups present within their polymerised structure [35,41–42]. Including (i) polyacids with weakly acidic groups (i.e., -COOH or -SO_3_H), (ii) polybases with weakly basic groups (i.e., -NH_2_), and (iii) polyamphoterics with both weakly acidic and weakly basic groups.

The scientific community is especially interested in using these properties in specific drug-delivery systems; the potential of using polysaccharide-based specific drug-delivery systems in the colon has been explored [43], as within the colon are many polysaccharides and a large number of bacteria which secrete enzymes [41]. Within this work, varying the concentration of various pH-sensitive polymers was considered in terms of swelling, and it was shown that abrupt changes in swelling could be obtained at a specific pH while using materials compatible with the colon. Also, assessing swelling as a function of NaCl concentration began to explore osmotic interactions of hydrogels, adding to the consideration of environmental conditions and providing data in a region often overlooked in these types of studies.

A great range of pH changes are faced when a foreign material travels through the human system. For example, for an orally administered drug-delivery device to release therapeutic agents in the colon, it must withstand a range of conditions as it passes through the body, including pH 6.2–7.3 in the mouth, pH 7 in the oesophageal tract, pH 1.5–3.5 in the stomach, pH 6 in the small intestine to pH 7.4 (terminal ileum) to 5.7 (caecum), and then a final pH 6.1–7.5 in the colon [44]. Often, in order for the therapeutic agent to reach the target site, specific barriers to variable pH must be designed for. Furthermore, a response to pH can be utilised to release the therapeutic agent at a site of specific acidity, thus ensuring targeted delivery.

Poly(acrylic acid)-based cryogels have been investigated as a pH oscillator in oscillatory bromate-sulphite-ferrocyanide reactions as potential soft materials for energy generation [45]. Boyaci and Orakdogen reported pH-responsive cryogels based on the monomer *N*,*N*-dimethylaminoethyl methacrylate (DMAEMA), crosslinked with acrylamido-2-methylpropanosulphonic acid (AMPS), where the swelling of the cryogels was heavily dependent on solution pH [46]. pH-Responsive cryogels have also been prepared comprising polyamidoamine (PAMAM) dendrimers, whereby the cryogels were stable under acidic conditions, and degrade at physiological solution pH (7.4) [47].

#### 3.3. Dual temperature and pH-responsive cryogels

During cryogel design and production a diverse range of starting monomers and/or polymers can be used, selected for desired properties to be exhibited in the final polymer matrix. Therefore, it is possible to combine thermo-responsive and pH-responsive properties through cryogelation techniques, resulting in a cryogel which responds to both changes in environmental temperature and pH. For example, grafting thermally responsive PNIPAM to a backbone of pH-responsive chitosan to produce a cryogel responsive to both temperature and pH has been explored in some detail [48–52], and the application as a drug-delivery system in the intestine has also been considered [12–13,34–35,42]. In particular, the work by Huang et al. demonstrated not only those systems responsive to both pH and temperature can be successfully produced using techniques which are scalable in theory to an industrial level, but also that these properties can be tailored to result in long-term release of therapeutic agents in environments modelling the human body [51]. A further example of multi-stimuli responsive cryogels includes the work by Dragan et al., who prepared semi-interpenetrating polymer networks (semi-IPN) hydrogels comprising DMAEMA and either potato starch or anionically modified polystyrene via a cryogelation procedure [53]. These materials were investigated as drug-delivery systems (DDS) and the release profile was strongly influenced by the pH. The authors suggested that due to a low release of the drug at pH 1.3, and an increase in release rate at pH 7.4, the material could be a potential for targeting release in the colon.

### 4. Other cryogel applications

For cryogel biomedical applications not discussed in section 5, including cell separation, tissue engineering scaffolds, bioreactors and capturing of target molecules, the reader is directed to a recent review by Bakhshpour et al. [54]. In addition to the biomedical applications discussed in detail below, cryogels have a variety of potential uses in fields such as tissue engineering [12], chromatography, and separation applications. For example, for the filtration of biologically relevant molecules [19,42], wastewater treatment [55–56], biosensors [57], as actuators [58–59], as carbon super-capacitators, anodic component of lithium-ion batteries, and devices for low-pressure H_2_ storage have also been explored [60].

### 5. Biomedical applications

Cryogels are of major interest in several fields of research, through offering new solutions and improvements to current systems and procedures. Their interconnected porosity in the micrometre scale, superior mechanical strength, and stability in comparison to hydrogels, thermodynamic compatibility with water (and thus also aqueous solutions), and being able to produce them from biocompatible materials make these materials ideal for cell culture and tissue engineering [12,16,38,42]. Furthermore, post-synthesis modifications can be performed to enhance attachment from certain objects, for example proteins from the extracellular matrix (ECM) in tissue engineering, cell culture and microbiology, or specific chemicals to aid in chemical, environmental, and medicinal filtration, and purification applications [5,19,42,60–61]. This also aids cell immobilisation, putting cryogels forward for potential use in bioreactors [5,42]. Cells can be included within the cryogel matrix, and shown benefits of doing this include reinforcing the matrix, increasing rigidity, and accelerating formation of pores [16,60,62].

Cryogels offer solutions to obstacles in current medicinal and therapeutic practices. In particular, specific isolation and characterisation of stem cells in cell-based therapies, detection of low levels of biomarkers in the blood (i.e., tumour cells, pathogenic microorganisms, etc.) for disease diagnosis, and general isolation of biological substances for clinical and environmental microbiology hold opportunities for cryogels [13,42,61]. They also allow for the processing of cell and virus suspensions and support microbiological research in studying interactions between different biological substances (i.e., cell–virus interactions) [38].

Highly dense polymeric structures in the walls result in cryogels having high elasticities, making them suitable for applications of a cyclic nature such as storage and sterilisation of biomedical materials, biocatalysts, bioreactors, actuators, biosensors, and more. They exhibit stability during repetitive freezing and thawing, and dehydrate/rehydrate and undergo cyclic compression without losing mechanical integrity [5,12–13,60]. Possessing shape memory allows for dehydration and storing; rehydration before use restores their original shape [37,60].

Here, we discuss emerging areas for cryogel application, including recent advancements in the use of cryogels in 3D printing, injectable cryogels, drug delivery and wound healing applications. It should be noted that whilst this section contains reference to tissue engineering, expansive detail on the subject matter is beyond the scope of this review.

#### 5.1. 3D-Bioprinting of cryogels

3D-printing of biomaterials, or bioprinting, enables the control of the size, porosity, and geometry of the final product tailored to the requirements of the individual patient, e.g., potential scaffold fabrication from cryogels in tissue engineering [63]. It is extremely important to consider the viscosity and injectability of the material for limitations on deposition mechanisms, e.g., the maximum deposition force and/or syringe tip size (0.8 mm used for hydrogels) for certain printers, place restrictions on highly viscous materials. These material properties have a direct influence on the final printing resolution. The resolution should be adequate for millimetre-sized defects (common in most in vivo tissue-engineering work in small animal models) [64].

Kim and co-workers reported a method in 2009, whereby a 3D-plotting system was coupled to a cryogenic refrigeration system [65–66]. Using these systems 3D scaffolds were prepared by printing collagen layers first on the cryogenic stage, where they froze immediately. To prevent clogging, the nozzle tip was covered with silicone rubber. The fabricated scaffolds were investigated with dermal applications in mind, and keratinocytes and fibroblasts were shown to migrate and differentiate within the scaffolds. They further reported the development of 3D-printed cryogels based on alginate with hierarchical structures for hard tissue applications [67].

More recently, in 2018 Serex et al. developed a microfabricated dispensing probe that allowed for the mixing of cryogel monomeric components immediately prior to printing [68]. They demonstrated the ability to control pore sizes by altering the temperature of the frozen bed used and the temperature of the dispensing probe. Successfully 3D-printed sodium carboxymethyl cellulose (CMC)-based cryogels were subsequently coated with collagen to promote cell adhesion. Cultivation and spreading of fibroblast NOR-10 cells were achieved within the cryogels, suggesting promise for tissue engineering applications.

**Figure 3:**
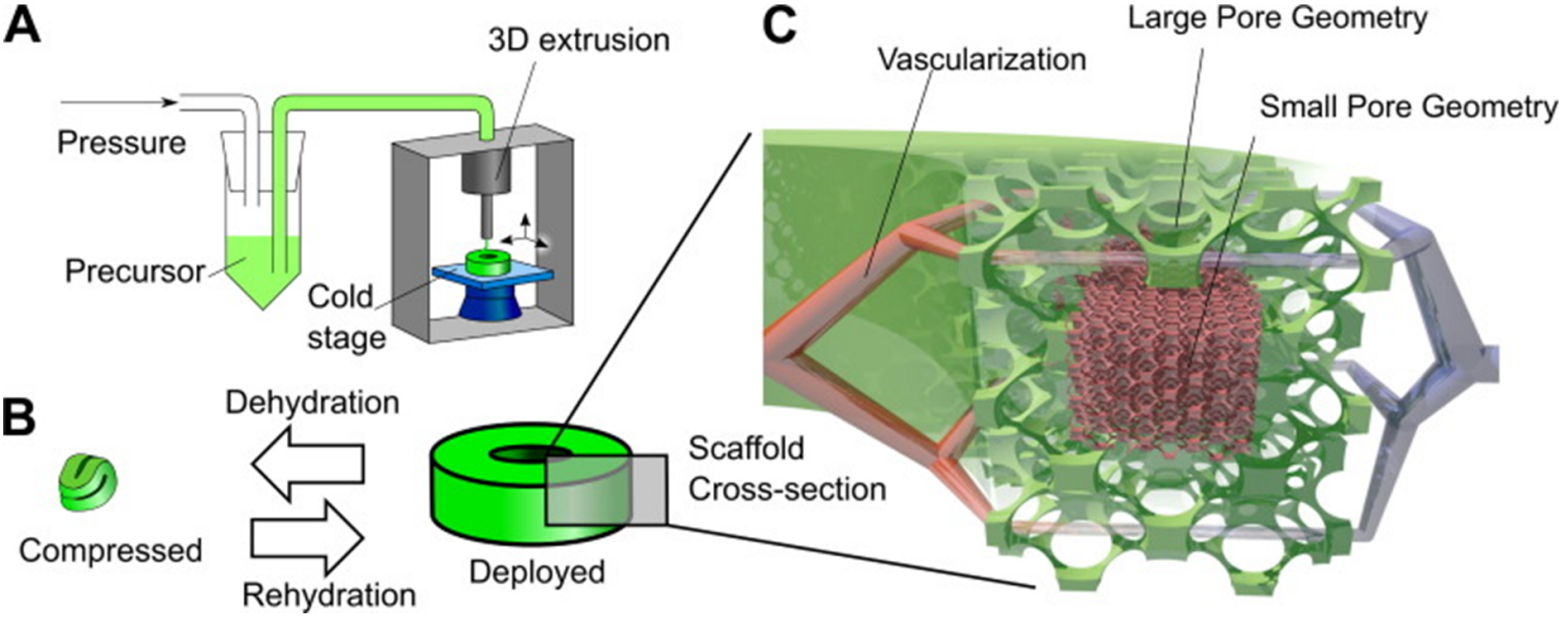
Principle of 3D-cryogel printing. A) Illustration of 3D-printing of cryogels. B) Illustration of the 4D shape change. By withdrawing fluid, the cryogel was highly dehydrated and compressed; in addition, surface tension drove surface-minimising folding. Upon rehydration, the cryogel returned to its original shape and volume via shape memory. C) Illustration of the hierarchical scaffold organisation. Local pore size variation was used to preferentially drive in vitro cellular organisation and in vivo vascularisation to areas with large pores. Reprinted from [69], Acta Biomaterialia, vol. 76, by A. Béduer; N. Piacentini; L. Aeberli; A. Da Silva; C. A. Verheyen; F. Bonini; A. Rochat; A. Filippova; L. Serex; P. Renaud; T. Braschler, “Additive manufacturing of hierarchical injectable scaffolds for tissue engineering”, 71–79, Copyright (2018) Acta Materialia Inc., with permission from Elsevier. This content is not subject to CC BY 4.0.

Building upon this work, hierarchical injectable cryogels were developed by Braschler and co-workers, based upon 3D additive manufacturing techniques [69]. They report 3D-printed structures which could be used for minimally invasive cell delivery (see Figure 3). Moreover, hierarchical structures with varying local pore sizes were obtained by tuning the substrate temperature during printing, which led to control over vascularisation density in vivo.

Biçen Ünlüer et al. recently reported the use of biocompatible gelatin–hyaluronic acid (Gel-HA)-based 3D-printed cryogels, which demonstrated biocompatibility without the need for additional coating [70]. In this work, the Gel-HA based bioink was 3D-printed to give a free-standing structure which was subsequently frozen to prepare the cryogel, prior to freeze drying. While this approach offers less control over the process than that reported by Serex, Braschler and co-workers, it does not require the cold stage necessary for cryogelation to occur during printing. Hybrid collagen/chitosan bioinks have also been investigated for their printability for producing cryogel scaffolds [71]. These cryogels form crosslinks through physical interactions, hence no additional crosslinking was required.

Some difficulties arise when designing 3D-printed cryogels. While precise resolution may be achieved, there is some difficulty maintaining porous structures when stacking multiple layers, specifically the fusion of layer contact points without scaffold collapse. The vertical pores remain intact, but horizontal pores in hydrogel scaffolds might collapse due to material softness [64]. Shick et al. also pointed out the limitation in fabricating scaffolds with high porosity and complex internal structures, for example in tissue engineering applications [63]. They report the processes being not yet developed enough to be used in clinical applications. However, with the use of novel materials and development of printing systems, progress can be made in achieving stable print structures for the ultra-high standards set by the human body.

#### 5.2. Injectable cryogels

Implantable biomaterials are proposed as a viable solution when dealing with issues relating to either the delivery or recruitment of cells. Currently the methods used are implantable biomaterials or polymeric hydrogels [18]. The issue with implantable biomaterials is that the implantation process requires the fabrication of the biomaterial component, trained physicians, post-operation patient distress, potential scarring, risk of infection, and often causes inflammation at the site of surgical incision. These issues may inhibit the performance of the implant [18] or cause distrust in the patient and the implant which may lead to negative emotional experiences [72]. Polymeric hydrogels, however, can cause issues of their own, such as the risk of liquid presursors leaking from the implant site to other tissues, and posing difficulties in generating the desired implant geometry [73]. An alternative proposal is the use of cryogels. This process typically results in a biomaterial with a unique macroporous network, shape-memory properties, and exceptional flexibility allowing syringe injectability [9].

It has been suggested that cryogels are more suitable for this application, as opposed to hydrogels, as a greater number of cells can be contained within the structure. With an exemption for injectable supramolecular hydrogel systems, which have been demonstrated to perform very well even after injection [74–76], hydrogels in general have been cited as being too brittle to enable injection [27]. Injectable cryogels have been investigated as scaffolds, for drug delivery, and for wound-healing applications. Here we highlight recent advances in the development of these injectable systems relevant to the applications discussed.

**5.2.1. Injectable cell scaffolding:** Injectable cryogels may be able to provide a suitable scaffolding for the attachment, proliferation, and survival of cells in complicated operations. The macroporous structure is an attractive option due to its low toxicity and to control the release of compounds stored in the porous structure through one of four methods: diffusion-controlled, swelling-controlled, erosion-controlled, and stimulus-controlled [54].

A study by Koshy et al. reported a methacrylated gelatin (GelMA) cryogel implanted by injecting through a conventional needle [18]. Gelatin was chosen for its inherent peptide sequences that facilitate cell adhesion and enzymatic degradation. Gelatin is also low cost and safe to use in human testing as shown in a work done by Nichol et al. [77]. The bulk mechanical behaviour, structure, and degradation of the cryogelated GelMA was tested, as well as the ability of these injected scaffolds to promote cell attachment, proliferation, and survival. Their results show that cryogels have a useful ability to retain their original shape even when extruded through a needle, this means that the cryogel does not leak out of the needle bore after being applied. The GelMA can then be implied with minimal intrusion to the patient which decrease the chance of trauma to the patient. Koshy et al. also report that in vitro and in vivo testing showed that cryogelated GelMA is cell and tissue compatible, although mild inflammation occurred during in vivo tests, however this is a common occurrence with biomaterials as shown by Mikos et al. [78]. Finally, the cryogel was capable of a controlled release of proteins. This allowed for a more controlled integration and movement of cells interconnecting with pores of the gel. These cell-attracting properties suggest that the use of cryogelated GelMA as a method for cell-integrated scaffolding and protein release for applications in biomaterials-based therapy is a viable option. Similar work was carried out by Lai et al. [79], whereby the polymerisation of GelMA was carried out using carboxybetaine methacrylate (CBMA) as a comonomer. With the incorporation of CBMA, these hydrogels demonstrated better mechanical properties, a slower degradation rate, and a controlled drug release rate compared with the GelMA alone. The properties of the GelMA/CBMA hydrogels could also be adjusted by varying the ratio of CBMA to GelMA which gives it a high degree of customisability.

The use of cryogels in neuroscience applications is gaining interest due to their soft and spongey properties [80]. Newland et al. recently reported on the use of injectable PEG/heparin-containing cryogels with nerve growth factor to promote the growth of neurite cells [81]. Through the use of template-assisted photopolymerisation, cylindrical cryogels were formed as high aspect ratios are desired for bridging across regions when whole neural pathways or large brain areas are targeted.

**5.2.2. Injectable nanocomposite cryogels:** Nanocomposite cryogels comprise a typical cryogel with nanoparticulate fillers incorporated into the structure. Addition of nanoparticles is often conducted to impart specific properties, such as improving mechanical properties or provide therapeutic properties such as antimicrobial properties. A work reported by Koshy et al. in 2018 was testing the use of injectable nanocomposite cryogels for versatile protein-drug delivery [37]. Injectable and porous cryogels were prepared using a bio-orthogonal click chemistry crosslinking approach with alginate employing tetrazine-norbornene coupling. Laponite nanoparticles were then incorporated within the walls of the cryogel. The results showed the so-called “click alginate” was able to produce cryogels with an interconnected porous structure, and high deformability allowing it to be used through a 16-gauge needle. The pore size range was between 50–300 μm, and small concentrations of laponite found in the gel wall helped with sustained release of a number of proteins with diverse properties [37].

**Figure 4:**
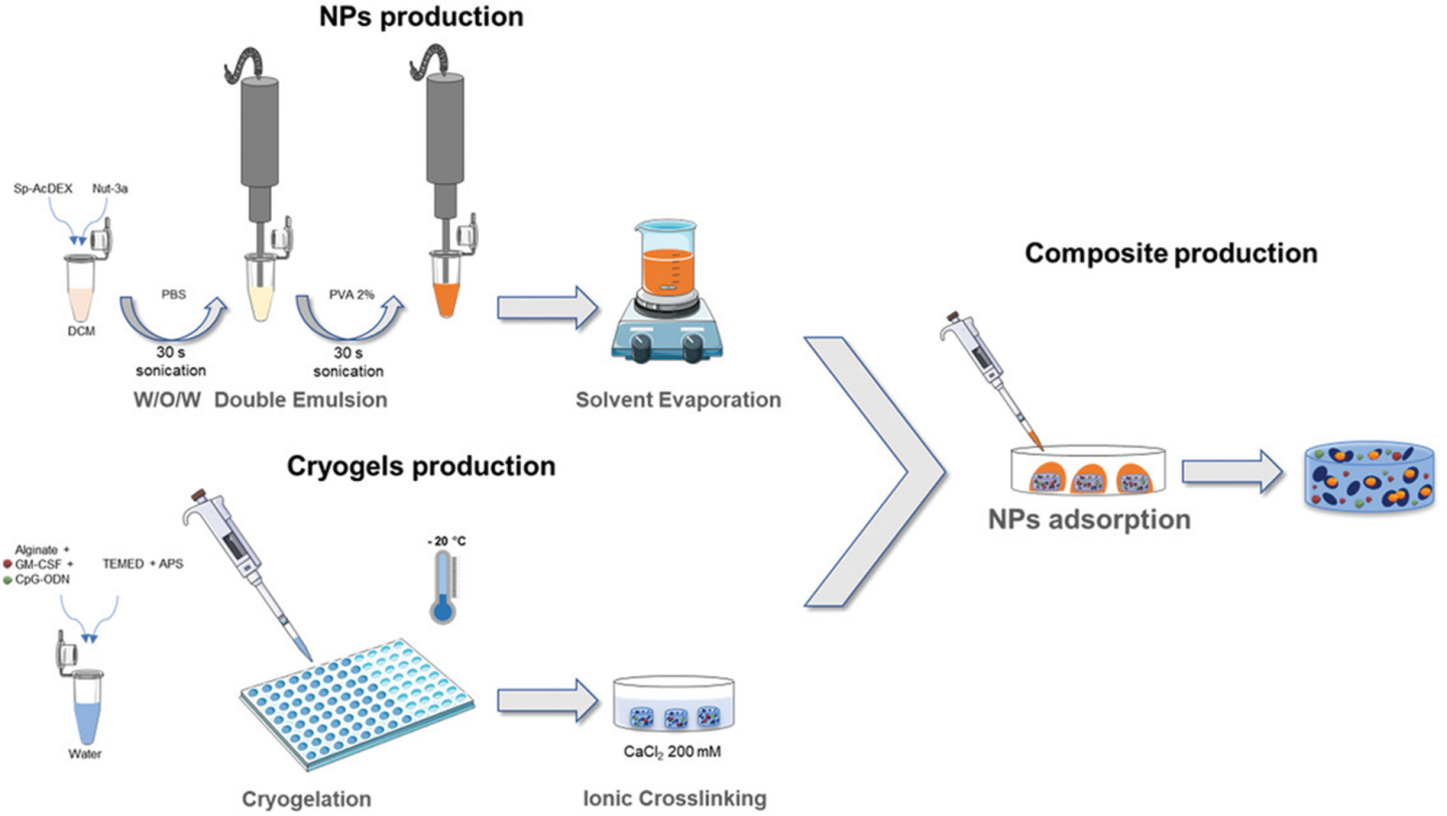
Illustration of the production of the injectable multifunctional composite, comprised of alginate cryogels loaded with GM-CSF and CpG-ODN and Sp-AcDEX NPs loaded with Nut-3a. Nut-3a loaded Sp-AcDEX NPs were produced by double emulsion technique and alginate cryogels were prepared by double crosslinking (covalent and ionic). The composite was prepared by allowing the Sp-AcDEX NPs to adsorb into freshly prepared alginate cryogels. Figure 4 was reproduced from [82], T. Bauleth-Ramos et al., “Acetalated Dextran Nanoparticles Loaded into an Injectable Alginate Cryogel for Combined Chemotherapy and Cancer Vaccination”, Adv. Funct. Mater., with permission from John Wiley and Sons. Copyright © 2019 WILEY-VCH Verlag GmbH & Co. KGaA, Weinheim. This content is not subject to CC BY 4.0.

Injectable nanocomposite cryogels have also appeared in research as a method to combat cancer. Bauleth-Ramos et al. used acetalated dextran nanoparticles in an injectable cryogel to combat the negative effects of chemotherapy and even suggested this approach as a potential method of vaccination (Figure 4) [82]. The injectable alginate cryogel was loaded with several therapeutic compounds, including spermine-modified acetalated dextran nanoparticles (Sp-AcDEX NPs). The Sp-AcDEX NPs were released over time to provide therapeutic effects with the nanoparticles aiding in accumulation in the tumour tissue. The reported results were found to be promising, as this method helped induce immunogenic cell death in tumour cells whilst also accumulating drug payloads into the tumour. Further testing was said to be targeted towards the use of the cryogel as an approach to delay or prevent cancer recurrence through the induction of in situ cancer vaccination mediated by antigens and danger signals released from the apoptotic cancer cells [82].

Additional to the work done by Bauleth-Ramos et al., a biomaterial-based vaccination system has been developed by Bencherif et al. [27]. It used minimal extracorporeal manipulation to provide in situ enhancement of dendritic cell (DC) numbers, a physical space where DCs interface with transplanted tumour cells, and an immunogenic additive. Cryogels were injected into mice to localise transplanted tumour cells and deliver immunomodulatory factors. After 4 days of cellular investigation, it was revealed that sponges loaded with 1.5 μg of granulocyte macrophage colony stimulating factor (GM-CSF) led to a significant increase in the total number of cells, and more particularly infiltration by DCs. The works done by Bauleth-Ramos et al. [82] and Bencherif et al. [27] suggest that the cryogels may help to combat against cancers.

Injectable composite cryogels based on GelMA, and cellulose nanocrystals (CNC) or polyamidoamine (PAMAM) dendrimers have been shown to be effective for minimally invasive drug delivery [83]. The reinforced cryogels main function was to provide scaffolding for cell growth and also incorporated the anti-inflammatory corticosteroid drug betamethasone sodium phosphate (BSP) for delivery when implanted. While optimum formulations were identified with the PAMAM dendrimer present, the release of BSP was relatively high within a 24-hour period, indicating that further development is needed to achieve sustained release with this system.

After reviewing the recent diverse work currently being achieved in the field of injectable cryogels we can identify that the use of cryogel is not only novel but also extremely effective and easily achievable. Potential risks arise in the use of injectable gels which occur in the presence of not fully polymerised gels being injected into patients. The non-polymerised precursors can cause free radical damage or react with proteins in the human body containing thiols and amino groups [84]. Further toxicity research would be required for all of these cryogels to fully understand how they react in the human body as opposed to animal substitutes.

However, the use of injectable cryogels is a subject matter that has yet to be fully studied. Use of hydrogels in these areas is well known, while cryogels offer most of the benefits that hydrogels can offer with a few added benefits such as their inherent porosity and open macroporous structure which can remain intact after injection [13]. The potential to incorporate nanoparticles within cryogels is also an area of interest due to the interesting properties and chemical interactions both materials have. Bauleth-Ramos et al. even went a step further to combine the nanoparticles healing effects with the cryogel sustained release formulation. Potential fields of study here could include the increase in injectable cryogels that guide drugs (due to the pinpoint injection method and chemical sensitivity [82]) and nanoparticles to targeted sites maximising the efficiency of healing in modern day medicine.

#### 5.3. Cryogels in drug delivery

Polymers are commonly used in drug-delivery applications as they can improve bioavailability of hydrophobic drugs and facilitate a controlled release of the drug. This leads to numerous benefits, including the increased time spent in the therapeutic zone, which allows the drug to be effective but is below the level considered toxic [85]. Drug-delivery systems (DDS) demand safe and effective treatment, and in the majority of cases a long-term target specific treatment would be favoured over general multi-dose pharmaceuticals. Cryogels have been identified as a good material of choice for the development of DDS, as properties can be tailored to meet the exact requirements necessary for a treatment. A major motivation for drug delivery is to produce a system that is a low-cost alternative to current methods, whilst retaining similar if not better performance. Cryogels’ inexpensive preparation and storage make them desirable for replacing currently used DDS where appropriate [73,82]. Moreover, stimuli-responsive cryogels can also be used to control drug release under specific conditions. There are also some emerging areas where cryogels are proving to be uniquely equipped for certain tasks due to their adaptability and physical-chemical properties [18].

Macroporous polymeric gels with their unique heterogeneous open-porous structure open new perspectives for the development of innovative systems for biomedical and pharmaceutical applications. Cryotropic gelation is an efficient method for the preparation of super-macroporous polymer hydrogels. They have large pore sizes which can easily contain samples and additives whilst also retaining biocompatible structures [86]. Some reports focus on the use of model drug compounds [87–89], or newly synthesised antimicrobial compounds [90], for example. Here, we highlight recent examples whereby a drug of clinical relevance has been investigated using cryogels for delivery.

**Figure 5:**
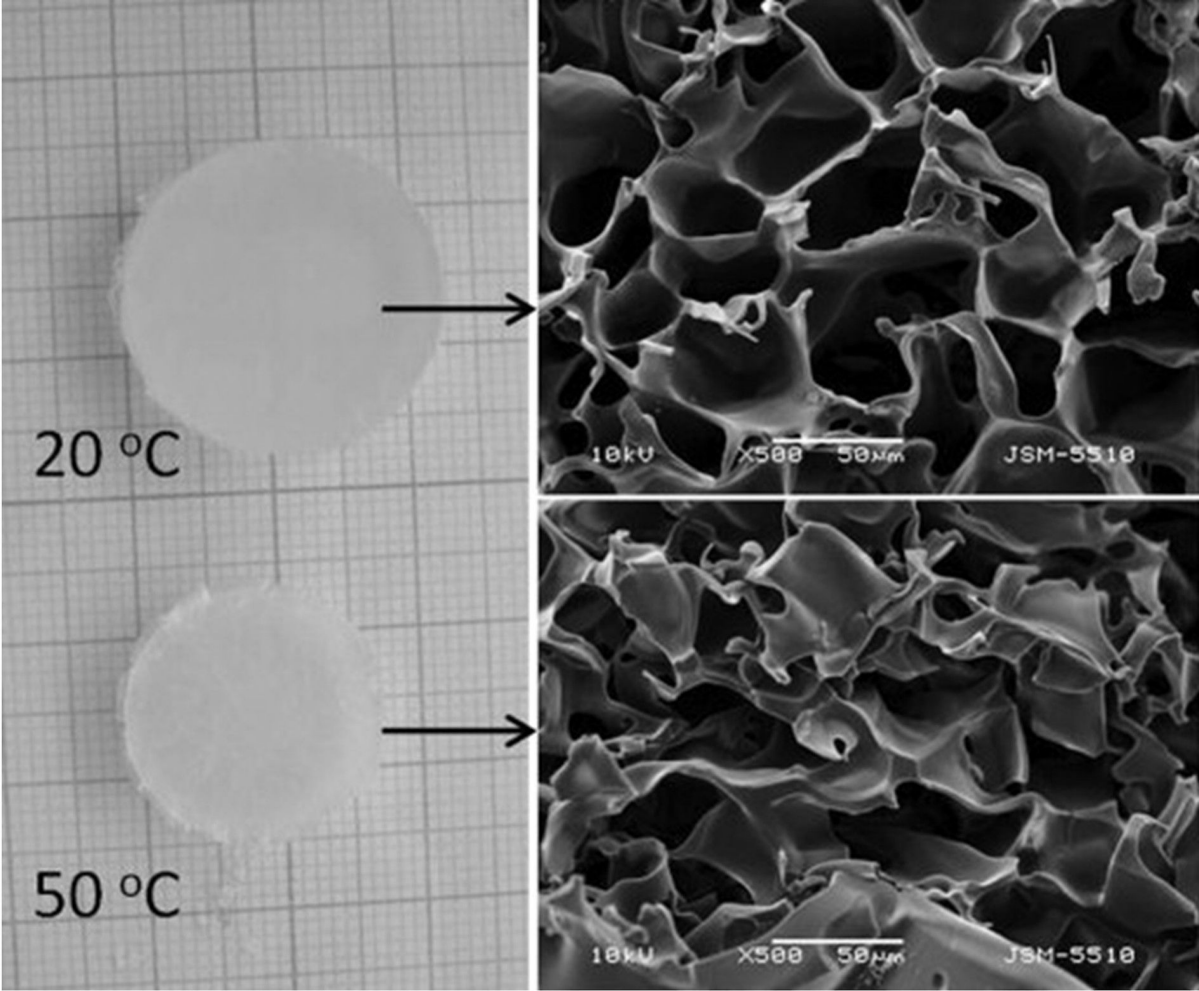
Digital and SEM photographs of PETEGA cryogel at 20 °C (top) and 50 °C (bottom), synthesised via UV irradiation of a moderately frozen system (10 mass % solution of ETEGA; temperature of freezing −20 °C; 5 mass % H_2_O_2_ and 30 mass % PEGDA to the monomer). Reprinted from [91], Polymer, vol. 52, issue 5, by B. Kostova; D. Momekova; P. Petrov; G. Momekov; N. Toncheva-Moncheva; C. B. Tsvetanov; N. Lambov, “Poly(ethoxytriethyleneglycol acrylate) cryogels as novel sustained drug release systems for oral application”, 1217–1222, Copyright (2011), with permission from Elsevier. This content is not subject to CC BY 4.0.

Kostova et al. published research on the novel approach of using cryogels with poly(ethoxytriethyleneglycol acrylate) (PETEGA) in 2011 (see Figure 5) [91]. They reported that the addition of the drug verapamil hydrochloride had no effect on the polymerisation of the cryogel, as gel yields close to 100% were obtained. The verapamil hydrochloride carried via PETEGA cryogels possessed sustained release over a period of more than 8 h, which is attributed to the hydrophobic state of the polymer network at physiological temperature and the method of drug immobilisation. Additionally, cryogels based on other polymers such as polyacrylamide (PAAm), PNIPAM and poly(2-hydroxyethyl methacrylate) (PHEMA), obtained via the same method reported by Kostava et al., have shown similar effects as reported by Petrov et al. in 2007 [30] and 2009 [92].

**Figure 6:**
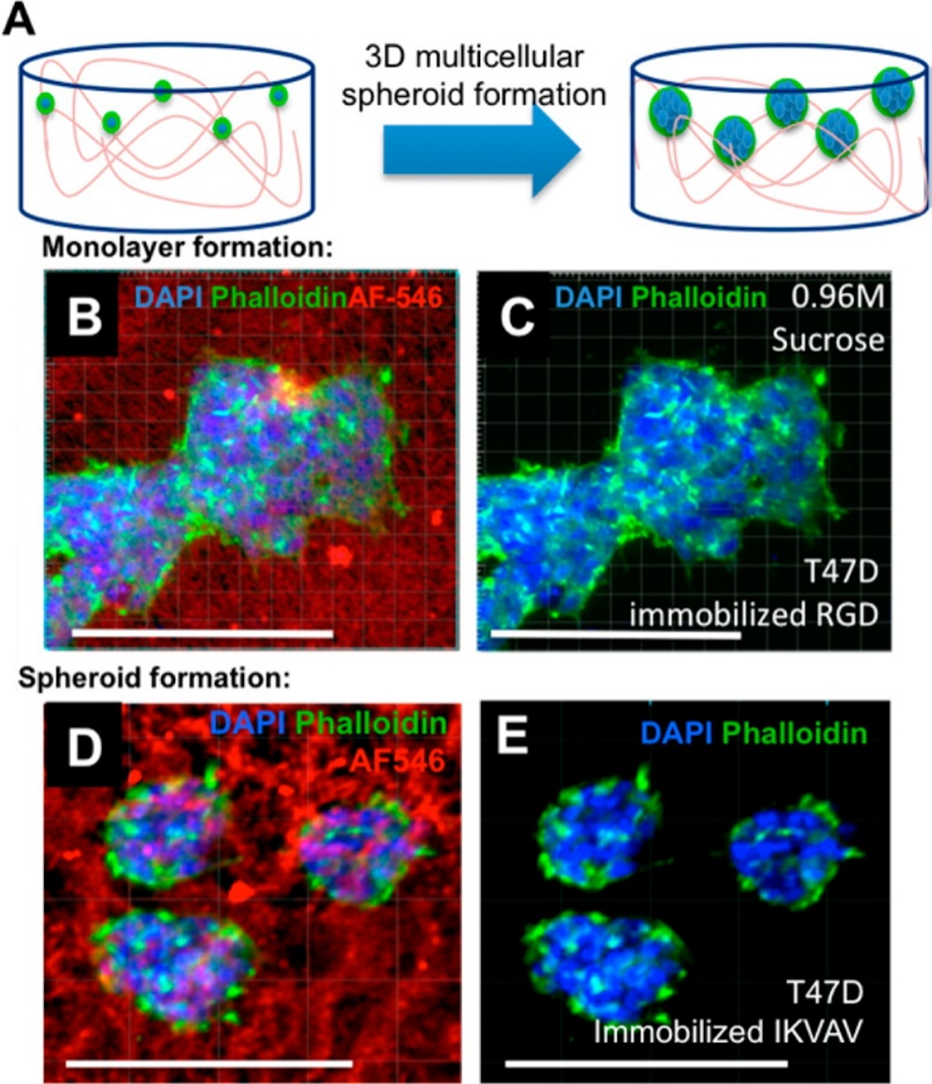
Cell morphology of T47D breast cancer cells cultured in HA cryogels. (A) Schematic representation of cells cultured on HA cryogels resulting in formation of 3D multicellular spheroids. Fluorescently labelled cryogels (red) immobilised with (B, C) RGD peptides result in cell monolayers whereas cryogels immobilised with (D, E) IKVAV peptides result in cellular spheroids. Scale bars represent 200 μm. Reprinted with permission from [94]. Copyright 2016 American Chemical Society. This content is not subject to CC BY 4.0.

More recently, cryogels have been used to tackle diseases such as cancer. Aliperta et al. have used cryogels in the sustained release of bispecific antibodies for cancer immunotherapy [93], combining stem cells with a biopolymeric cryogel DDS. Human mesenchymal stromal cells (MSCs) were gene-modified to secrete anti-CD33-anti-CD3 bispecific antibodies (bsAb) to be used for the treatment of acute myeloid leukaemia (AML). Macroporous star-shaped poly(ethylene glycol) (starPEG)-heparin cryogels were prepared by combining hydrogel network formation via chemical cross-linking of starPEG and heparin at 4 °C and pH 8. The initial tests were reported to be a success with rapid and efficient transport of nutrients and therapeutic bsAbs via the interconnected macropores, however, more testing is required on its effects on long lasting T cells. Similar positive results were attained by Tam et al., who synthesized biomimetic cryogels whilst also analysing the mechanism of their formation [7,94]. They examined the effects of mono/disaccharide additives on the size of pores and how they interact with polysaccharide polymers to alter cryogel pore size and mechanical properties. In addition, Tam et al. demonstrated the optical transparency with three-dimensional spatial control of immobilised bioactive growth factors using multiphoton patterning and cellular response to immobilised ligands as shown in Figure 6 [94].

Researchers are working on the design of effective and low-cost gel-based DDS. Biopolymeric porous gels can be derived from natural materials such as chitosan. Dinu et al. prepared macroporous structures based on chitosan and clinoptilolite by cryogelation [95]. The group investigated and tested their potential as a DDS using diclofenac sodium and indomethacin as the drugs. They reported the cumulative release of diclofenac sodium from the monoliths lower than 5% at pH 1.2 and higher than 70% at pH 7.4. In addition, they reported cumulative release 6% of indomethacin within the first hour in phosphate buffered saline (PBS) from composite cryogels.

Lima et al. presented an interesting partnership of cryogels with ceramics for a DDS [96]. Polyvinyl alcohol and polyacrylic acid were used as the precursor to the cryogel, and integrated within the ceramic. They produced a layer-by-layer structure by adding both cryogels and ceramics into a mould, and a physical cross-linker was used to realise the drug theophylline after stimuli had been achieved. Due to the layer system, drug release could occur without diffusion of the subsequent layers, controlling the speed of the release in the ceramic cryogel. In addition, the ceramic can enhance the mechanical properties of composite within the cryogel and can be tailored for speciﬁc drugs and drug-release proﬁles [96].

**Figure 7:**
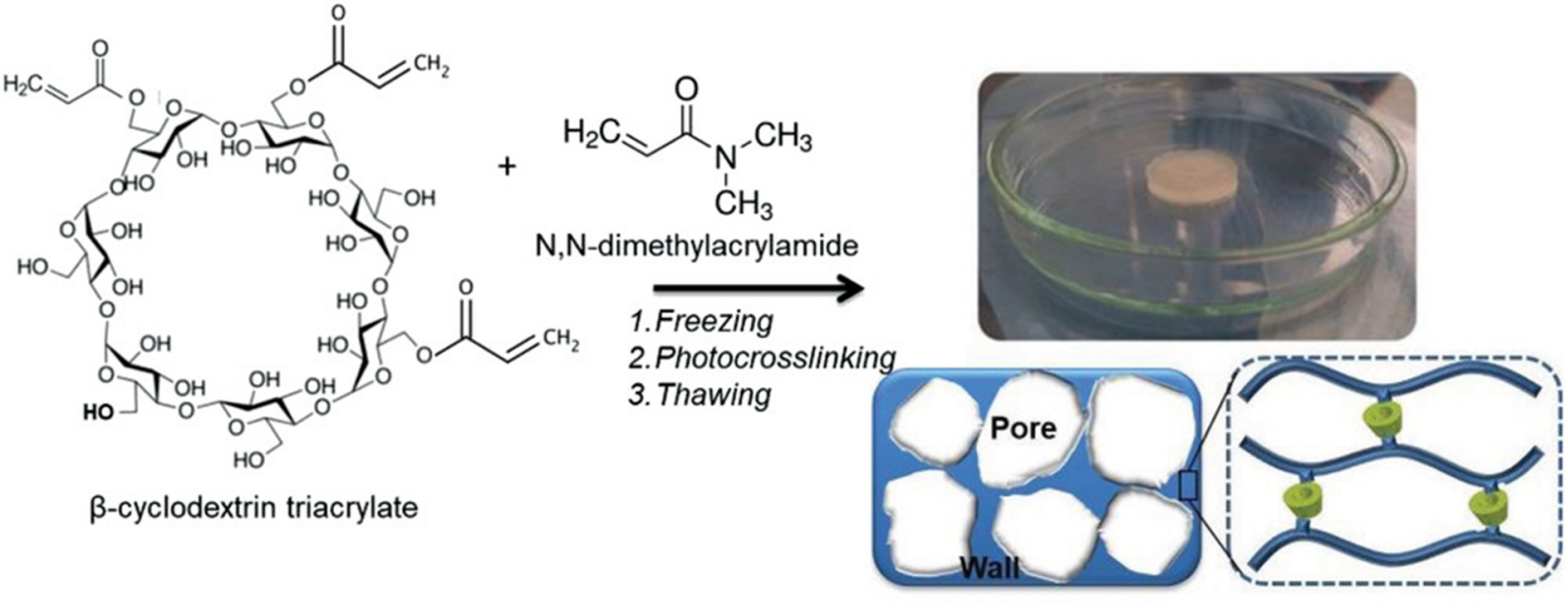
Preparation of PDMA/β-CD cryogel via cryogenic treatment and photochemical crosslinking in frozen state. Figure 7 was reproduced from [97], Y. Danov et al., “Cryogel Carriers Comprising β‐Cyclodextrin Moieties for Improved Solubilization and Delivery of Aripiprazole”, Macromol. Chem. Phys., with permission from John Wiley and Sons. Copyright © 2021 Wiley-VCH GmbH. This content is not subject to CC BY 4.0.

The drug aripiprazole used to treat schizophrenia was recently investigated for delivery using cryogels comprising β-cyclodextrin (β-CD) functionality [97]. The cryogels were synthesised by photochemical crosslinking of *N*,*N*-dimethylacrylamide (DMA) and β-CD triacrylate (Figure 7) and aripiprazole was incorporated through inclusion inside the hydrophobic β*-*CD domains. The release of aripiprazole was monitored at pH 1.2 and 6.8, targeting oral delivery, with different release profiles depending on the ratio of DMA/β*-*CD.

The antifungal agent voriconazole was incorporated into a physically crosslinked cryogel comprising PVA and chitosan grafted with NIPAAm (CS-*g*-PNIPAM) for mucosal applications [98]. Cell culture assays confirmed that the cryogels were non-toxic and the release profiles of voriconazole from a CS-*g*-PNIPAM/PVA 75/25 gel showed release of up to 80% of the encapsulated drug over a period of 8 hours. Sustained release of diclofenac over two weeks was studied using natural biopolymer kefiran cryogels [99]. Kefiran is a biocompatible water-soluble branched polysaccharide, isolated from kefir grains. Scaffolds were prepared by cryogelation of a 2% w/v aqueous solution of the kefiran, without the need for any crosslinking agents. Release of diclofenac, a non-steroidal anti-inflammatory drug, was low with 15% of the drug released after 2 weeks. However, this could result in a sustained release over longer time periods which would require further investigation. Doxorubicin release from heparin-containing cryogel microcarriers was investigated by Newland et al. [100]. Highly sulphated heparin was used to exploit electrostatic interactions between anionic sulphate groups and the primary amine group present in doxorubicin which confers a positive charge under physiological conditions. This interaction was confirmed by in silico modelling. While the carriers did not show any cytotoxicity, cell viability was reduced in the presence of the doxorubicin-loaded cryogels, suggesting delivery of the drug was successful. These cryogels were also injected into mice, adjacent to an orthotopic breast cancer tumour, impeding tumour growth and metastasis.

Several groups have reported on the use of composite cryogels for drug delivery, whereby the active ingredient is encapsulated within a particle, such as polymeric micelles [101] or microneedles [102], embedded within the cryogel. Therapeutic agents may not necessarily be represented solely by drug molecules. Recent examples of other types of therapeutic cargos used for delivery by cryogels include fertilizers for agrochemical applications [103], peptides [104], proteins [37], and growth factors [81,105–106]. For example, Lee et al. reported the use of a double cryogel structure for the delivery of growth factor for enhanced bone regeneration [105].

#### 5.4. Cryogels in wound healing

The skin acts as a protective barrier against the environment with immunologic and sensorial functions [7]. As of now, the way of dealing with extensive skin loss would be wound dressing, autografts, and allographs but there is a lot of room for improvement. Cryogels can cause cell migration which shows promise to solving these problems and enhancing skin substitutes [7]. An optimal bio-scaffold would have to be biocompatible, biodegradable, have a high pore connectivity and swelling ratio as its function is to promote cell growth and act as a nucleus for cell migration. Moreover, ideally it would also promote hemostasis, the physiological process that stops bleeding. Certain cryogels have the potential to provide all the ideal criteria listed above; this is brought about by careful consideration of the material chemistry and processing techniques. Pore connectivity is needed as it facilitates metabolic and oxygen transport. When cryogels are fully hydrated, they often exhibit a soft consistency which in turn creates low interfacial tension. This low interfacial tension minimises irritation to surrounding tissue post-implantation. This theory has been put into practice because Priya et al, investigated the ability of cryogels to mimic various layers of skin [107]. A polyvinylpyrrolidone-iodine cryogel was used as the top layer to impart antiseptic properties, while the bottom regenerative layer comprised a gelatin cryogel. When the cryogel had been implanted into rabbits which had sustained wounds, the animals with cryogels implanted showed faster and more productive wound healing compared to the untreated rabbits and a complete skin regeneration occurred after 4 weeks with no inflammatory response.

**Figure 8:**
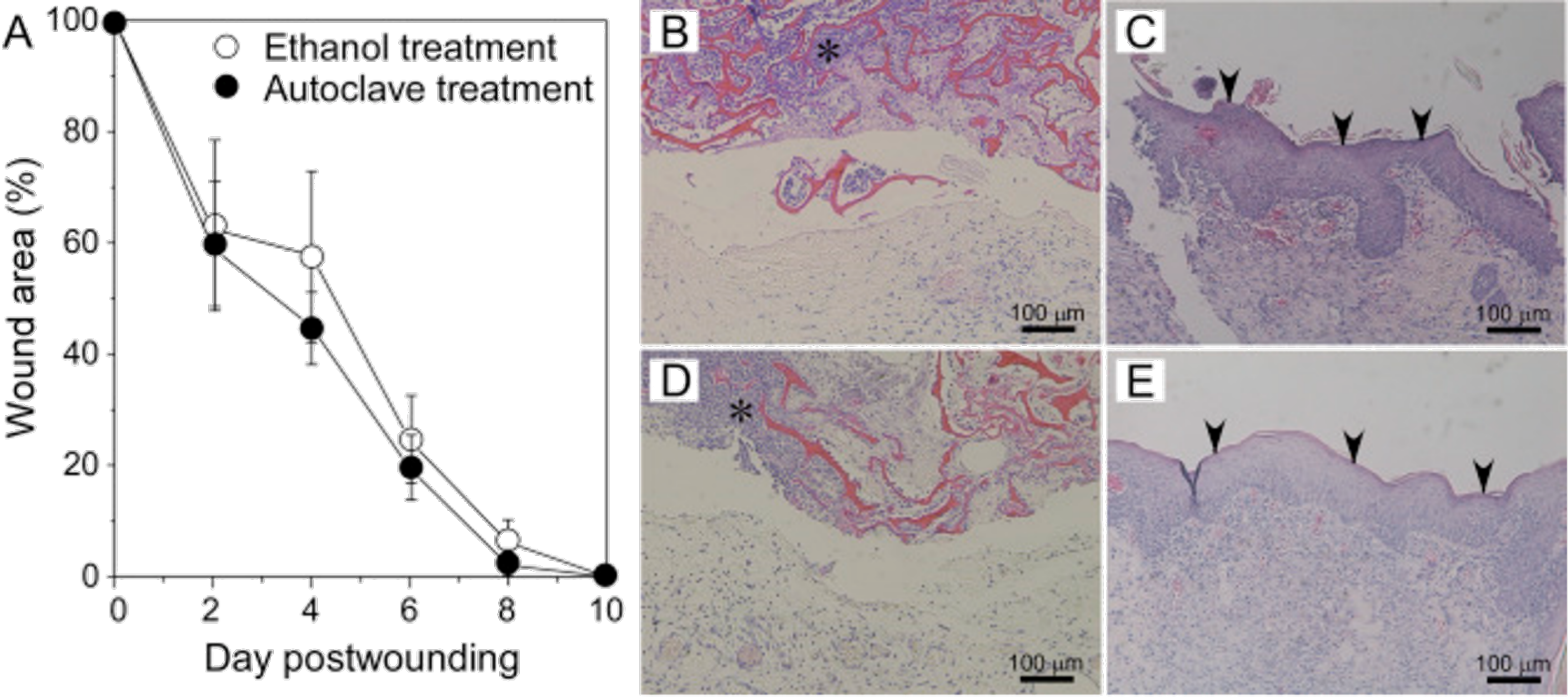
(A) Healing rate of wounds treated with autoclaved CG11 cryogels and those treated with 70% ethanol. (B–E) Hematoxylin and eosin-stained sections of wound tissues treated with autoclaved CG11 cryogels (B, C) and those treated with 70% ethanol (D, E) at 2 days (B, D) and 10 days post wounding (C, E). Reprinted from [115], Journal of Bioscience and Bioengineering, vol. 125, issue 4, by T. Takei; S. Danjo; S. Sakoguchi; S. Tanaka; T. Yoshinaga; H. Nishimata; M. Yoshida, “Autoclavable physically-crosslinked chitosan cryogel as a wound dressing”, 490–495, Copyright (2018) The Society for Biotechnology, Japan, with permission from Elsevier. This content is not subject to CC BY 4.0.

Most reports of cryogels used in wound-healing applications use a naturally occurring biopolymer such as gelatin and/or chitosan in combination with other components. Gelatin and chitosan are known to be biocompatible [108–109], and while gelatin is known to promote cell adhesion [108], chitosan owns haemostatic properties [109]. Neres Santos et al. produced PVA/gelatin cryogels incorporated with manuka honey in the matrix for wound-healing applications [110]. The manuka honey was used to diffuse to the wound through controlled release from the cryogel matrix. Gelatin has also been suggested to be used as scaffolding material in absorbent pads for wound dressing and for surgical use [111]. Chhatri et al. developed physically crosslinked PVA/chitosan cryogels loaded with Savlon (antiseptic liquid) and suggested wound-healing applications for the material [112]. This theory is supported by literature that chitosan is known to accelerate the wound healing process in humans and providing antibacterial properties as well [113]. Physically crosslinked chitosan-gluconic acid cryogels have also been reported by Takei et al., who investigated applying differing methods of sterilisation, using either ethanol washing [114], or by autoclaving the cryogels [115]. Interestingly, the cryogels retained their haemostatic properties following autoclaving at 121 °C for 20 min, and showed comparable healing rates compared to ethanol sterilisation (Figure 8).

**Figure 9:**
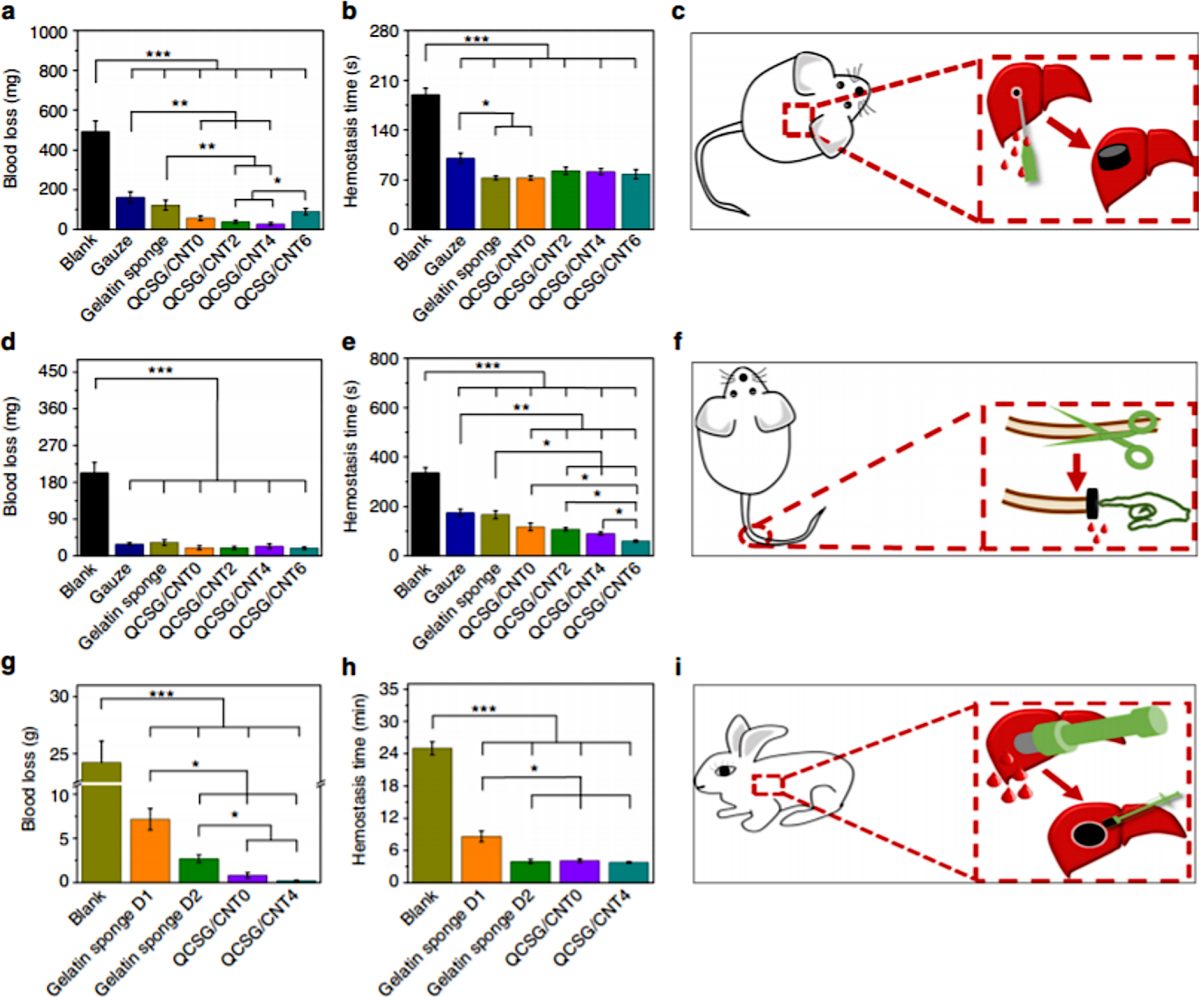
In vivo haemostatic capacity evaluation of the cryogels. Blood loss (a) and haemostatic time (b) in the mouse liver injury model. Schematic (c) represents the injury model for the mouse. Blood loss (d) and haemostatic time (e) in the mouse-tail amputation model. Schematic (f) represents the amputation model of the mouse tail. Blood loss (g) and haemostatic time (h) in the rabbit liver defect lethal noncompressible haemorrhage model. Schematic (i) represents the noncompressible haemorrhage model of the rabbit’s liver. Reproduced from [116] (Copyright © 2018 X. Zhao et al., distributed under the terms of the Creative Commons Attribution 4.0 International License, https://creativecommons.org/licenses/by/4.0/).

A unique take to the development of cryogels as injectable gels is the work achieved by Zhao et al. in developing an injectable antibacterial and conductive shape memory haemostatic cryogel [116]. The cryogel is based on carbon nanotubes (CNT) combined with methacrylated chitosan. Zhao et al. report that the cryogel has robust mechanical strength which is required to aid in wound healing. The shape recovery mechanism of the cryogel is also triggered by blood and has high absorption speed and uptake values, this would mean the cryogel works well for blood clotting purposes. Overall, it was tested against gelatin sponges and traditional gauze which it outperformed. Figure 9 shows the results of Zhao et al. in vivo experimentation. The effectiveness of the CNT-reinforced cryogel can be seen as it consistently is shown outperforming the gauze and/or gelatin sponges. The results concluded that cryogel QCSG/CNT4 was shown to have better haemostatic capability than QCSG/CNT0, and better in vivo wound-healing performance than Tegaderm™ film, gelatin sponge, gauze, and QCSG/CNT0 [116].

Cryogels comprising the natural biopolymers silk fibroin and chitosan, with tannic acid and ferric ions (Fe^3+^) incorporated have been reported as multifunctional devices for wound healing providing hemostasis, exudate absorbance, antibacterial effects, and promotion of cell proliferation [117]. The tannic acid and ferric ions provide photothermal properties whereby under near infrared (NIR) radiation the temperature increased to enhance the antimicrobial properties, while also showing good hemostasis properties such as blood absorption and clotting, and cell proliferation. Chitosan has also been used by Meena et al. for producing cryogels for haemostatic applications, with locust bean gum (LBG) incorporated to enhance mechanical properties and water absorption [32]. This chitosan/LBG semi-interpenetrating network cryogel was investigated for its swelling, degradation, and protein adsorption properties, demonstrating potential as a haemostatic dressing.

The healing of wounds to skin tissue takes place over a much shorter time scale than the regeneration of bone tissue in fractures and breaks [118–119]. It has been reported that the majority of skin tissue wounding will be healed up to the proliferative phase by 4–6 days depending on the size, location, and severity of the wound [120]. The application of a degradable cryogel with a higher rate of degradation may be preferable compared to the rate of degradation that would be required for other purposes such as severe tissue engineering or bone tissue applications. However, it has been suggested that for wound dressing applications degradation of the cryogels is not wanted, as it may result in small pieces of cryogel remaining in the wound [121]. If full degradation of the cryogel was to occur, this would not be a problem.

## Conclusion

While cryogels have been investigated by researchers for decades, they are now finding applications in a broad range of biomedical settings due to their interconnected porosity and advantageous properties. A major advantage of cryogels is their low-cost manufacturing due to the medium of porogens commonly used being water, and relatively low amounts of reagent required. However, there are issues when considering scaling up cryogel syntheses.

The variability of cryogel compositions, dictated by the precursors used, as well as physical properties such as porosity, pore size distribution, wall thickness, and density have given researchers a plethora of tools with which to tune cryogels properties for separation, tissue engineering, for preparing printable and injectable cryogels, wound healing and drug delivery applications. Here, we have reviewed recent advances in the development of injectable cryogels and 3D printing with cryogels, and cryogel applications in wound healing and drug delivery. Showing that while 3D printing of cryogels is still in its infancy developments are being made to develop complex hierarchical structures, with potential to successfully mimic biological structures. This could have a huge impact on how we design scaffolds for tissue engineering and wound healing in the future. Furthermore, injectable cryogels have been an area of intense research in recent years, and injectables could pave the way for new and improved therapies with minimal invasive procedures for patients. Due to the structure of cryogels, they inherently have a high surface area and hence are a logical candidate for the release of therapeutics from the walls of the material. Similarly, they are excellent candidates for wound healing due to the ability of cryogels to swell and absorb water and provide a sterile environment for new tissue growth, supported by the porous structure.

However, after reviewing these areas of interest there is room for further investigation. For example, cryogels used in injectable gels for DDS is currently an emerging field. In the examples given previously there were no two methods that were similar and when authors compared the use of cryogels, there was mostly comparison to hydrogels rather than previously reported work involving cryogels. In the field of cryogels for drug delivery and wound healing there are several examples of systems that have demonstrated proof of principle. However, there are limited efforts dedicated to testing these on human test subjects which is required to learn the effects these potential drugs can have on humans whether long term or short term.

In the authors opinion, there is a huge positive outlook for the use of cryogels combatting cancer. Future research would be required to identify types of cancers that could be successfully treated using alternative methods such as cryogels, as opposed to well-known chemotherapies. Injectable nanocomposite cryogels have possible ties in cancer research and the potential to form vaccination against several types of cancer. More broadly, this platform may be useful for generating a range of T-cell effect responses from immunity to tolerance. The overall outlook for cryogels is incredibly positive and exciting as they are a subset of materials which may have a huge impact in many areas of biomedical science and medicine, which we hope to see implemented in the coming years.
